# Exosomal circPOLK promotes metastasis of NSCLC cells via regulating mir-1204/SOX8 axis

**DOI:** 10.1186/s12935-026-04306-2

**Published:** 2026-05-08

**Authors:** Yang-ling Li, Ye-han Liu, Jing Cheng, Zhen-qiang Wei, Neng-ming Lin, Chong Zhang

**Affiliations:** 1 Department of Clinical Pharmacology, Key Laboratory of Clinical Cancer Pharmacology and Toxicology Research of Zhejiang Province, Affiliated Hangzhou First People’s Hospital, School of Medicine, Westlake University, Hangzhou, 310006 Zhejiang China; 2 School of Medicine, Hangzhou City University, No. 51 Huzhou Street, Hangzhou, 310015 Zhejiang China; 3 College of Pharmaceutical Sciences, Zhejiang University, Hangzhou, 310058 Zhejiang China; 4 Department of Cardiothoracic Surgery, Affiliated Hangzhou First People’s Hospital, School of Medicine, Westlake University, Hangzhou, 310006 China

**Keywords:** Hsa_circ_0073052, NSCLC, Metastasis, Angiogenesis, MiR-1204, SOX8

## Abstract

Non-small cell lung cancer (NSCLC) is the most common subtype of lung cancer, and the prognosis of NSCLC patients is still poor particularly for patients with metastatic disease. Thus, extending the knowledge about the mechanism of metastasis is critical for NSCLC treatment. We demonstrated that hsa_circ_0073052 (circPOLK) was overexpressed in blood exosomes from cancer patients than those from healthy people. CircPOLK overexpression enhanced the metastasis and EMT progression of NSCLC cells. Next, circPOLK might function as miRNA sponge in NSCLC cells. Through bioinformatical prediction and RNA pull-down experiment, miR-1204 might be a potential target of circPOLK. Interestingly, serumal miR-1204 was an efficacious diagnostic and prognostic molecular for lung cancer patients and acted as a suppressor on NSCLC progression. Furthermore, circPOLK promoted metastasis of NSCLC via regulating miR-1204. Furthermore, SOX8 was identified as a potential target of circPOLK/miR-1204. GO enrichment of circPOLK regulated DEGs showed that circPOLK might be involved in angiogenesis of NSCLC. Indeed, circPOLK secreted by NSCLC cells could promote angiogenesis. Our dada not only identifies a novel circPOLK/miR-1204/SOX8 signaling pathway, but also provides therapeutical strategies for NSCLC patients with metastatic disease.

**Graphical abstract:**

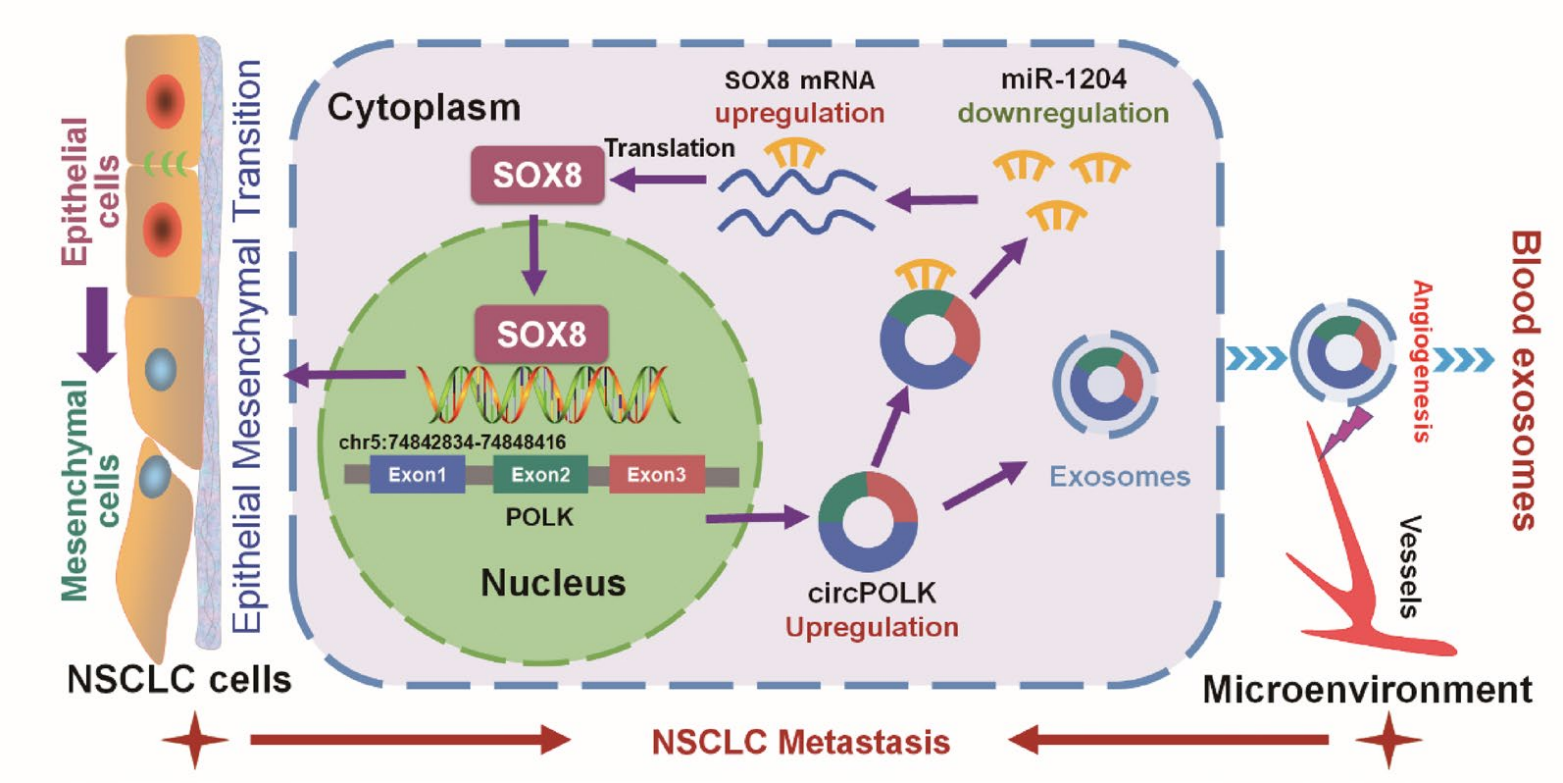

**Supplementary Information:**

The online version contains supplementary material available at 10.1186/s12935-026-04306-2.

## Introduction

Lung cancer is the leading cause of death worldwide, and non-small cell lung cancer (NSCLC) accounts for 85% of all lung cancer cases [1–4]. Despite the improvements in diagnosis and treatment of lung cancer, the prognosis of NSCLC patients remains unsatisfactory [5, 6]. The prognosis of NSCLC patients with different clinical stages is remarkably different [7]. Approximately 75% of patients present with locally advanced or even metastatic stage when they are diagnosed, and 5-year survive rate is merely 15% [8]. However, the 5-year survival of early-stage NSCLC patients can exceed 90%, and early diagnosis and therapy are immensely vital for NSCLC patients [9].

Exosomes are small extracellular vesicles with a size of 30–100 nm in diameter secreted by multiple cell types [10]. Exosomes contain a variety of functional molecules, including lipids, proteins, and nucleic acids allowing cell-to-cell communication [11]. These molecules are specifically delivered to the neighboring or even distant cells far away from their release and reprogram the recipient cells [12]. They also exist in a wide array of bodily fluids, such as peripheral blood, ascites, urine [13]. Tumor-derived exosomes act an important role in regulating cancer development via cell communication in tumor microenvironment (TME) [14]. Besides, these exosomes shed into body fluids and are being studied as diagnostic, prognostic, predictive cancer biomarkers, or even therapeutical targets [15].

Circular RNA (circRNA), a type of covalently closed non-coding RNA, regulates gene expression in eukaryotes. Compared with other non-coding RNAs, circRNA is stronger stability due to the covalently closed structure which prevents from exonuclease-regulated degradation [16, 17]. Meanwhile, circRNAs have been detected in exosomes and abundant in several body fluids such as blood and saliva [18]. Therefore, circRNAs have advantages over other non-coding RNAs as molecular diagnostic markers and therapeutical targets [19]. Furthermore, exosomal circRNAs regulate multiple processes in cancer progression such as angiogenesis, therapeutical resistance and metastasis [20–22]. Multiple circRNAs in tumor-derived exosomes play important roles in metastasis via promoting cancer cell invasion and angiogenesis in TME [23]. In this study, we first identified the existence of hsa_circ_0073052, which is referred to as circPOLK hereafter, in human blood exosomes, and circPOLK promoted metastasis via regulating mir-1204/SOX8 axis in NSCLC cells. Furthermore, exosomal circPOLK enhanced angiogenesis via regulating TME. Thus, this study not only revealed the role and mechanism of circPOLK in NSCLC progression but also verified a potential application of exosomal circPOLK as innovative disease biomarker.

## Materials and methods

### Materials

Antibodies against E-cadherin (cat. 20874-1-AP), SOX8 (cat. 20627-1-AP), and N-cadherin (cat. 22018-1-AP) were supplied by Proteintech. Antibody against Snail (cat. 3879 S) was obtained from Cell Signaling Technology. Anti-argonaute-2 antibody (AGO2; cat. ab186733) was purchased from Abcam. Anti-GAPDH antibody (cat. db106) was supplied by Diagbio.

### Cell culture

Human normal lung epithelial cells (BEAS-2B), human NSCLC cells (A549, NCI-H1299, NCI-H460), and human umbilical vein endothelial cells (HUVEC) were obtained from Shanghai Institute of Biochemistry and Cell Biology [5, 24], and they are cultured using 90% RPMI-1640 plus 10% FBS. The cell lines used in this study were mycoplasma-free cells and has been proven by DNA profiling within the last three years.

### Plasmid construction and cell transfection

Cells were transfected with siRNAs, microRNAs (miRNAs) or plasmids using jetPRIME. siRNA targeting circPOLK and SOX8, miR-1204 mimics and inhibitors, and negative controls were synthesized by GenePharma, and the sequences were showed in Table S1. The circPOLK shRNA was cloned into pLKO.1-TRC empty vector. The circPOLK overexpressing plasmid was constructed by pLC5-ciR. The sequences of amplification primers were listed in Table S2.

### Quantitative reverse transcription-PCR (qRT-PCR) and genomic DNA extraction

Total RNA was extracted and transcribed into cDNA, and ChamQ SYBR qPCR Master Mix (Vazyme, Nanjing, China) was used for qRT-PCR. Genomic DNA (gDNA) was extracted using Easy Cell Genomic DNA Extraction Kit (Zhejiang Easydo Biotechnology Co.Ltd, China). All primers were showed in Table S2.

### RNase R and actinomycin D treatment

Total RNA (2 µg) was treated with 3 U/mg of RNase R for 30 min at 37 °C. The expression of circPOLK and its linear gene POLK were determined using qRT-PCR. The stability of circPOLK and its linear gene POLK were determined using qRT-PCR in NSCLC cells after the treatment of actinomycin D or DMSO.

### Fluorescence in situ hybridization (FISH)

FISH was performed to determine the subcellular localization of circPOLK. Briefly, NSCLC cells grown on coverslips were fixed with 4% paraformaldehyde, permeabilized with 0.1% Triton X‑100, and pre-hybridized. Cy3‑labeled probes targeting circPOLK (GenePharma, China) were then hybridized at 37 °C in a humidified chamber. After stringent washes to remove nonspecific binding, nuclei were counterstained with DAPI. Images were captured using a fluorescence/confocal microscope under identical settings [25]. The probe sequences are listed in Table S1.

### Wound healing assay

NSCLC cells (1 × 10^5^ cells/well) were treated with circPOLK siRNA or plasmid for 24 h, and 5 µM mitomycin C was used to suppress cell proliferation after pretreatment for 3 h. A wound was created by a sterile 10-µL pipette tip, and the cell images were recorded after 24 h [26].

### Transwell migration and invasion assay

Cell migration and invasion were assessed using 8-µm Transwell chambers. For invasion assays, inserts were precoated with Matrigel; for migration assays, Matrigel was not used. Cells were suspended in serum-free medium and seeded into the upper chambers (4 × 10^4^ cells for migration and 8 × 10^4^ cells for invasion), while medium containing serum was added to the lower chambers as a chemoattractant. After 24 h incubation, cells remaining on the upper surface were removed, and cells that migrated/invaded to the underside were fixed, stained, imaged, and counted in several randomly selected fields [27]. All experiments were performed in triplicate.

### Western blot analysis

20 µg protein lysates were separated and transferred to polyvinylidene fluoride membranes [28]. The membranes were blocked using 5% skim milk, and then treated with primary antibodies (overnight, 4 °C) and secondary antibodies (1.5 h, room temperature), and finally the bands were visualized [29].

### RNA sequence

Total RNA was extracted from NSCLC cells transfected with empty vector or circPOLK overexpression plasmid. RNA quality was evaluated, and qualified samples were used for library construction and sequenced on an Illumina platform by LC-Bio Technologies (Hangzhou, Zhejiang, China). Raw reads were quality-filtered and mapped to the human reference genome. Gene expression was quantified and differentially expressed genes were identified using an established pipeline (FDR < 0.05).

### Luciferase reporter assay

Dual-Luciferase Reporter Assay kit (Promega, USA) was applied to determine the interaction between circPOLK/miR-1204 or miR-1204/SOX8 mRNA. Wild-type (WT) of circPOLK was cloned into the firefly-tagged pGL4.21 promoter luciferase vector (Promega, USA). Mutant of circPOLK, WT of SOX8 and mutant of SOX8 were constructed by Loche (Hangzhou, China). Cells were transfected with miR-1204 mimic, circPOLK or its mutant plasmid using Lipofectamine 3000 [30]. After 48 h transfection, cells were lysed and fluorescence intensity was assayed by a dual luciferase assay system (BioTek, USA). The sequences of amplification primers were showed in Table S2.

### RNA immunoprecipitation assay (RIP) and RNA pull-down assay

A RIP kit (Geneseed, Guangzhou, China) was used to determine the interaction between AGO2 and circPOLK. RNA pull-down assay was determined by RNA Antisense Purification (RAP) Kit (BersinBio, China). The probe sequences are listed in Table S1.

### In vivo experiment

BALB/c nude mice were obtained from SLAC (Shanghai, China). NCI-H1299 cells were transfected with PLKO or shcircPOLK plasmid for 48 h, and injected into the tail veins of nude mice (1 × 10^6^ cells per mouse, *n* = 6 per group). After 60 days, the nude mice were sacrificed and the lung and liver metastasis were detected by staining with Bouin’s and haematoxylin and eosin staining separately.

### Statistical analysis

All data were presented as the mean ± SD. At least three biological replicates were used to compare the difference between groups. Two-tailed student’s t-test was used to examine the statistical analyses, and the difference was considered significant at *p < 0.05 [31] **p < 0.01, ***p < 0.001, ns, not significant. All experiments had been repeated at least three times.

## Results

### Identification and characterization of circPOLK in NSCLC cells

**Fig. 1:**
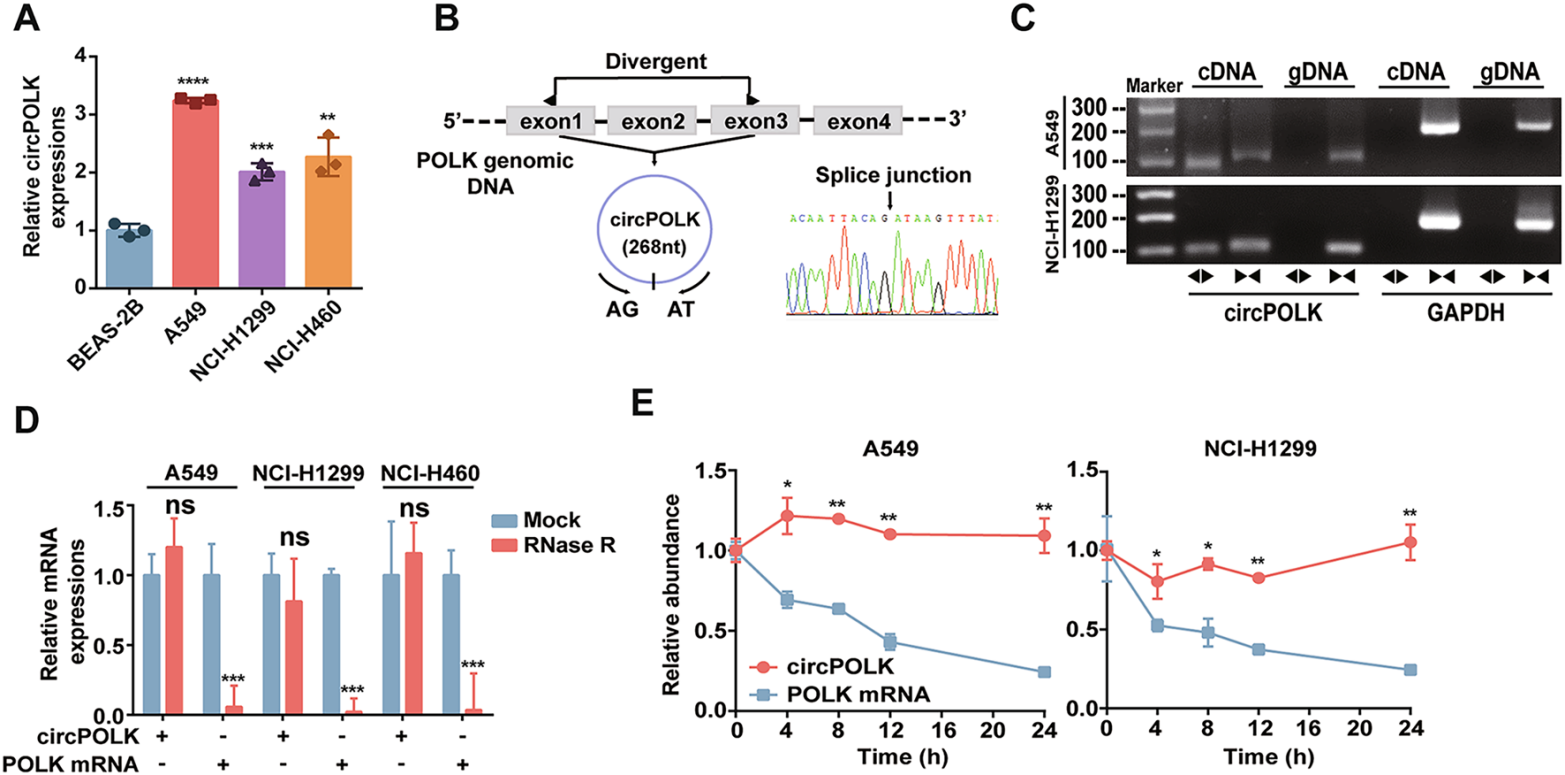
Identification and characterization of circPOLK in NSCLC cells. (**A**) qRT-PCR analysis of circPOLK in normal lung epithelial cells and NSCLC cells. (**B**) Schematic illustration indicating the generation of circPOLK from its host gene, and validation by Sanger sequencing in NCI-H1299 cells. (**C**) The cDNA and gDNA were collected from NSCLC cells, and combining PCR with an electrophoresis assay was used to amplify circPOLK using divergent primers and POLK using convergent primers in A549 and NCI-H1299 cells. (**D**) qRT-PCR analysis for the resistance of circPOLK and linear POLK to RNase R in A549, NCI-H1299 and NCI-H460 cells. (**E**) NSCLC cells were seeded in 6-well plates at a density of 1 × 10^5^/well, after 24 h, cells were treated with actinomycin D (2.0 mg/L) for the indicated times, and qRT-PCR was used to determine the abundance of circPOLK and POLK mRNA in A549 and NCI-H1299 cells

We collected blood exosomes from cancer patients and healthy people, and RNA sequencing results revealed that circPOLK was remarkably overexpressed in blood exosomes from cancer patients than those from healthy people (Fold change = 9.18, *p* = 1.45e-4; Figure S1A and 1B, Table S3) [32, 33]. Meanwhile, the level of circPOLK was also higher in NSCLC cells than in normal lung epithelial cells (Fig. 1A). Sanger sequencing verified the presence of circPOLK head-to-tail junctions and its generation from host gene POLK in NSCLC cells (Fig. 1B). Furthermore, circPOLK could be amplified using divergent primers by complementary DNA (cDNA) but failed to be amplified from genomic DNA (gDNA), and host gene POLK could be amplified by convergent primers using cDNA or gDNA in NSCLC cells (Fig. 1C). These data proved the existence of circPOLK in NSCLC cells. Subsequently, the POLK mRNA could be degraded by RNase R, but circPOLK was resistant to the digestion of RNase R (Fig. 1D). In addition, actinomycin D were applied to suppress new RNA synthesis to determine the stability of circPOLK. As shown in Fig. 1E, circPOLK was more stable than linear POLK mRNA in NSCLC cells. Thus, we prove the existence of circPOLK in NSCLC cells, and circPOLK is a circular RNA and generates from host gene POLK by back-splicing.

### circPOLK reinforces the metastasis of NSCLC cells

**Fig. 2:**
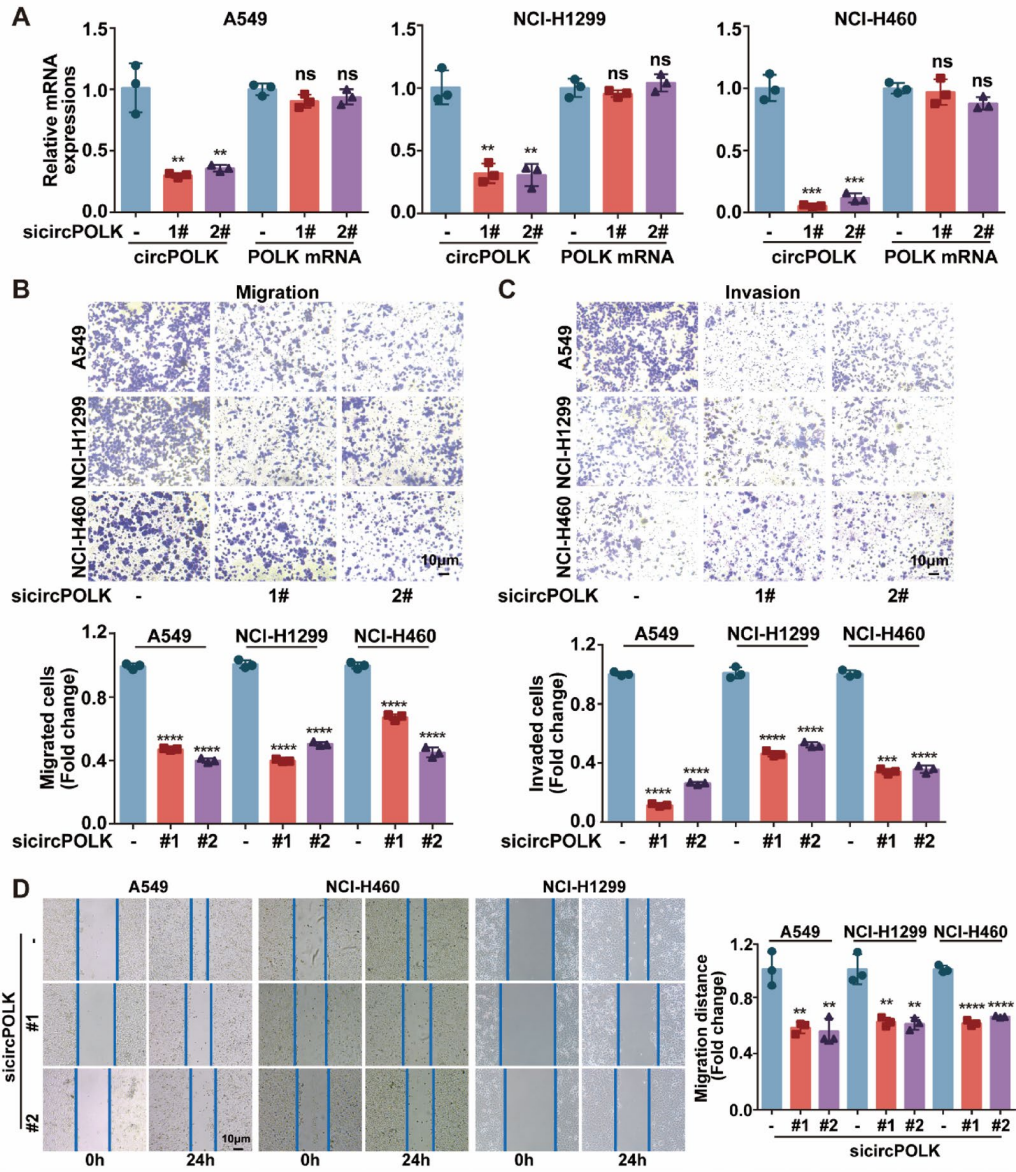
Knockdown of circPOLK inhibits the migration and invasion of NSCLC cells. (**A**) NSCLC cells were transfected with sicircPOLK and control siRNA for 48 h, and the expression of circPOLK and POLK mRNA were assessed by qRT-PCR in NSCLC cells. (**B**-**C**) NSCLC cells were transfected with sicircPOLK and control siRNA for 48 h, and the cell migration and invasion abilities were measured using Transwell assays. Scale bar: 10 μm. (**D**) NSCLC cells were transfected with sicircPOLK and control siRNA for 48 h, and the migratory capabilities of NSCLC cells were measured using wound healing assay. Scale bar: 10 μm

**Fig. 3:**
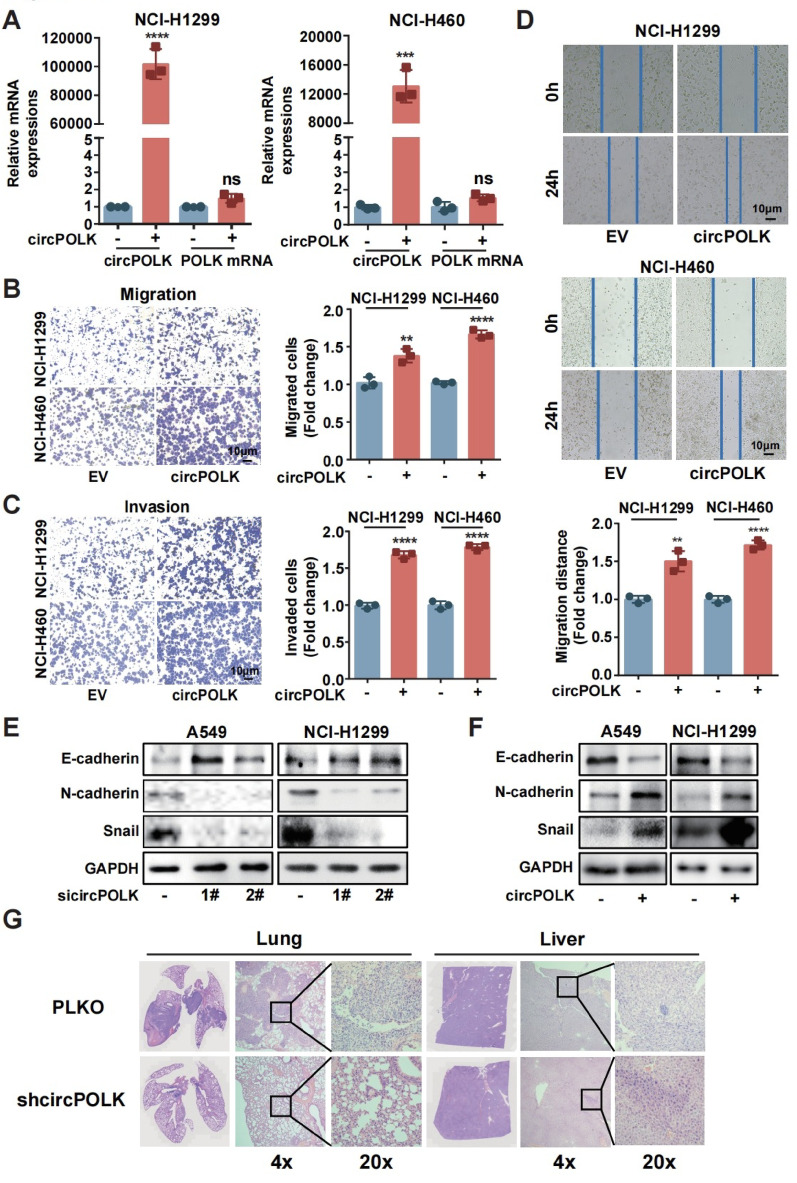
circPOLK promotes the metastasis of NSCLC cells in vitro and in vivo. (**A**) NSCLC cells were transfected with circPOLK overexpressing plasmid and empty vector for 48 h, and the expression of circPOLK and POLK mRNA were assessed by qRT-PCR in NCI-H1299 and NCI-H460 cells. (**B**-**C**) NSCLC cells were transfected with circPOLK overexpressing plasmid and empty vector for 48 h, and the cell migration and invasion were measured using Transwell. Scale bar: 10 μm. (**D**) NSCLC cells were transfected with circPOLK overexpressing plasmid and empty vector for 48 h, and the migration capability of NSCLC cells was determined by wound healing assay. Scale bar: 10 μm. (**E**-**F**) NSCLC cells were transfected with indicated siRNAs or plasmids for 48 h, and the expression of indicated proteins were determined by western blot analysis. (**G**) H&E staining of lung and liver metastatic tumors. The images were photographed at 4 × or 20 × magnification

Then, we explored the function of circPOLK in NSCLC progression, we silenced the expression of circPOLK using sicircPOLK targeting head-to-tail junctions without affecting the level of POLK (Fig. 2A). As shown in Figure S1A circPOLK knockdown could not affect the proliferation of NSCLC cells. However, circPOLK silence statistically inhibited the migration and invasion in NSCLC cells (Fig. 2B and C). Similarly, would healing assay also supported that the knockdown of circPOLK restrained the migratory abilities of NSCLC cells (Fig. 2D). Subsequently, we constructed the circPOLK overexpressing plasmid, and the level of circPOLK was statistically upregulated in NSCLC cells transfected with circPOLK overexpressing plasmid compared with empty vector (Fig. 3A). In contrast, circPOLK overexpression statistically augmented the migratory and invasive capacities of NSCLC cells (Fig. 3B and C). Meanwhile, the ectopic expression of circPOLK could remarkably promote the migratory capacity of NSCLC cells as demonstrated by wound healing assay (Fig. 3D). Furthermore, sicircPOLK suppressed the EMT progression of via increasing E-cadherin and suppressing N-cadherin, and circPOLK overexpression reinforced the EMT progression of NSCLC cells (Fig. 3E and F). Additionally, NSCLC transfected with shcircPOLK exhibited less metastatic foci in the livers or lungs of nude mice as compared to NSCLC cells transfected with empty vector (Fig. 3G and Figure S2).

### circPOLK functions as a sponge for miR-1204 in NSCLC cells

**Fig. 4:**
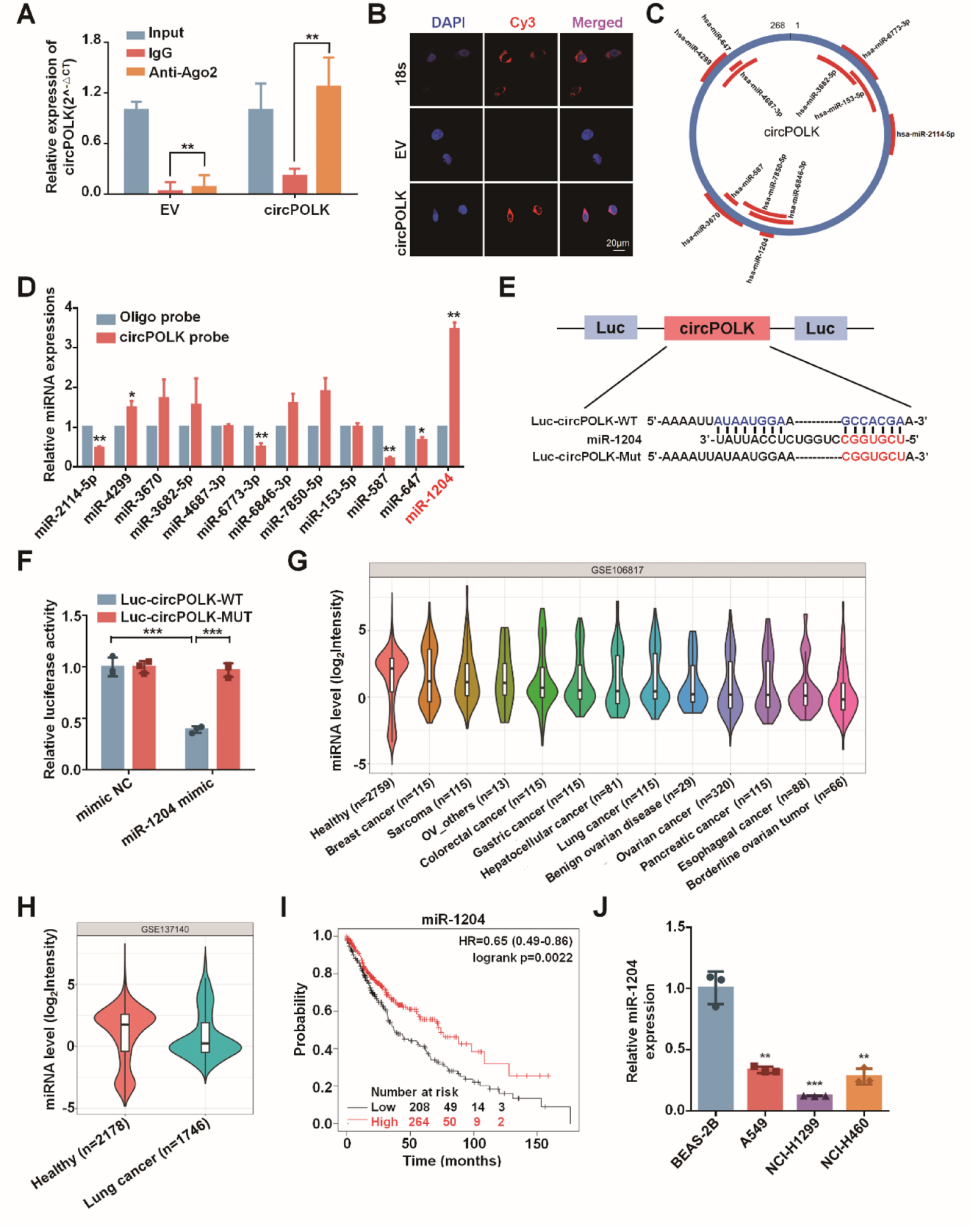
circPOLK serves as a sponge for miR-1204 in NSCLC cells. (**A**) The interaction between circPOLK and AGO2 was verified by RIP assay and qRT-PCR. (**B**) RNA FISH detecting circPOLK subcellular localization in NCI-H1299 cells. The circPOLK probe was labeled with Cy3 (red) and nuclei was stained with DAPI (blue). Scale bar: 20 μm. (**C**) Schematic illustration showing potential target miRNA of circPOLK as predicted by miRanda, RNA hybrid and circinteractome. (**D**) circPOLK was enriched by circPOLK FISH probe using RNA pull-down experiment, and subsequently qRT-PCR analysis was used to detect the expression of 12 potential target miRNAs sponged by circPOLK in NCI-H1299 cells. (**E**) A schema of circPOLK wild-type (WT) and mutant (Mut) luciferase reporter vectors. (**F**) Luciferase activity was tested in NCI-H1299 cells co-transfected with luciferase reporter containing circPOLK sequences with wild type and mutant binding site of miR-1204 and the mimic of miR-1204 or control. (**G**) The data was collected from CancerMIRNome database (http://bioinfo.jialab-ucr.org/CancerMIRNome/). Circulating miRNA expression; Dataset: GSE106817; Title: Integrated extracellular microRNA profiling for ovarian cancer screening Serum; *n* = 4046. (**H**) CancerMIRNome database analysis of miR-1204 expression in healthy and lung cancer patients. Dataset: GSE137140; Title: Blood test using serum microRNAs can discriminate lung cancer from non-cancer Serum; *n* = 3924. (**I**) The data was collected from Kaplan-Meier Plotter database (https://kmplot.com/analysis/). Gene symbol: miR-1204; Lung adenocarcinoma (*n* = 472). (**J**) qRT-PCR analysis detected the expression of miR-1204 in human normal lung epithelial cells and NSCLC cells

We next investigated the mechanism of circPOLK-mediated NSCLC metastasis. circRNA frequently functions as a miRNA sponge and regulate the levels of target genes. Thus, we first hypothesized that circPOLK might promote metastasis of NSCLC cells via sponging miRNA. Indeed, circPOLK was remarkably enriched by the AGO2 antibody compared with IgG using RIP assay, indicating that circPOLK might function as a binding platform between AGO2 and miRNAs (Fig. 4A). Furthermore, FISH analysis also demonstrated that circPOLK mostly located in the cytoplasm of NSCLC cells (Fig. 4B). We next predicted the potential target miRNAs of circPOLK based on miRanda, RNA hybrid, and circinteractome [34–36]. Through bioinformatical prediction programs, there were 12 overlapping target miRNAs of circPOLK (Table S4 and Fig. 4C). To further verify the potential targets of circPOLK, we established RNA pull-down to enrich circPOLK and its interacting partners. miR-4299 and miR-1204 could be statistically pulled down by circPOLK probe, and miR-1204 was efficiently pulled down by circPOLK in NCI-H1299 cells (Fig. 4D). The interaction between circPOLK and miR-1204 was predicted by circinteractome, and circPOLK and its mutant sequences were cloned as shown in Fig. 4E. Luciferase reporter assay showed that miR-1204 mimic could significantly sponge wild type circPOLK, but not mutant circPOLK (Fig. 4F). Furthermore, the level of extracellular miR-1204 was lower in the serum from multiple types of cancer patients than healthy persons (Fig. 4G) [37, 38]. Meanwhile, the expression of circulating miR-1204 was found to be significantly decreased in the serum of individuals diagnosed with lung cancer compared to that observed in healthy individuals (Fig. 4H) [39]. Additionally, low expression of miR-1204 predicted poor outcomes of lung cancer patients (Fig. 4I) [40]. Furthermore, the level of miR-1204 reduced in NSCLC cells compared to normal lung epithelial cells (Fig. 4J). Therefore, these data indicate that circPOLK may sponge miR-1204 in NSCLC cells, and serumal miR-1204 maybe a potential molecular marker for the diagnosis and prognosis of individuals with lung cancer.

### circPOLK promotes the metastatic capabilities of NSCLC through miR-1204

**Fig. 5:**
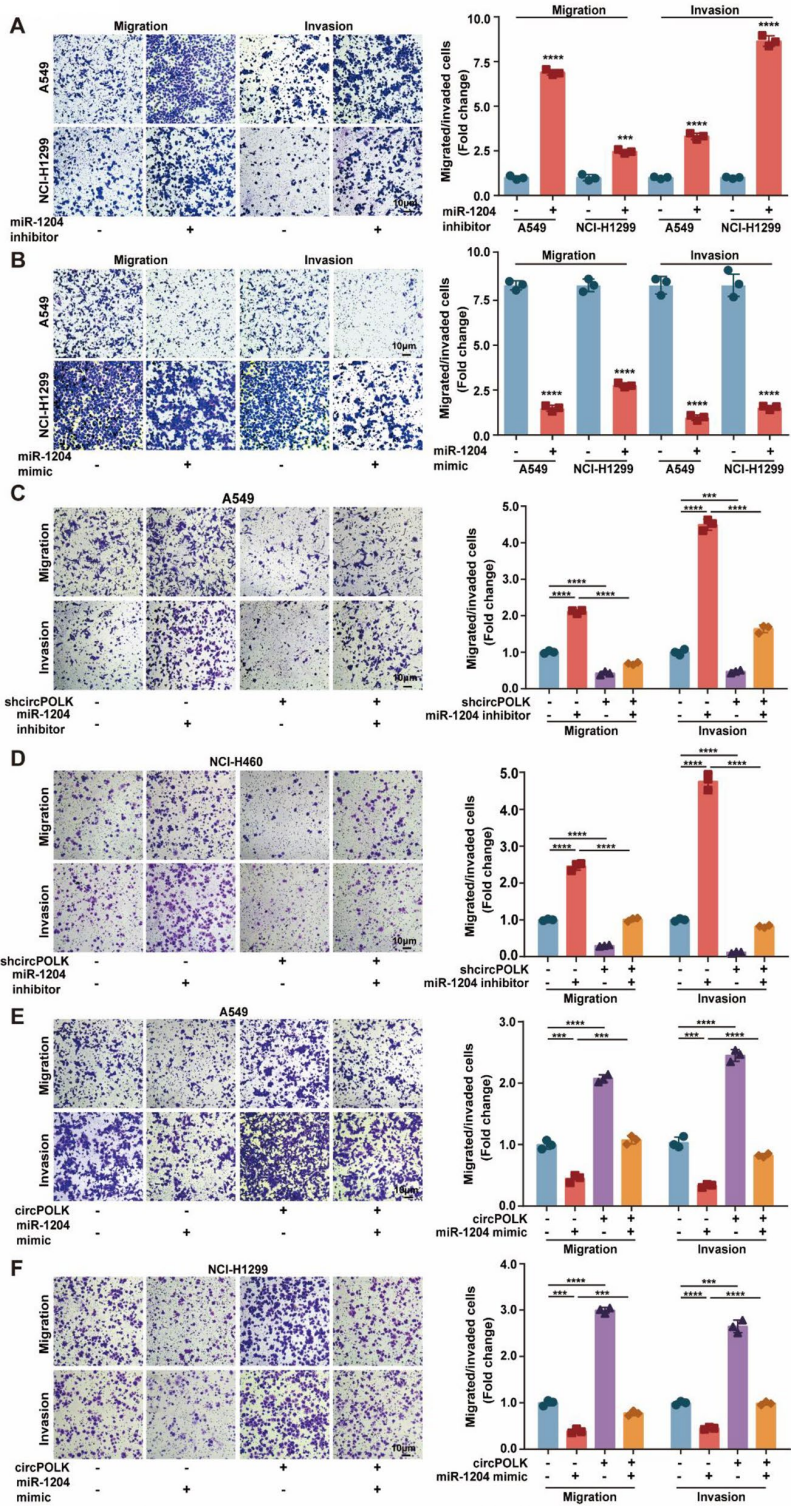
circPOLK promotes the metastatic potential of NSCLC cells by miR-1204. (**A**-**B**) NSCLC cells were transfected with miR-1204 inhibitor or mimic for 48 h, and the migratory and invasive abilities of NSCLC cells were determined by Transwell assay. Scale bar: 10 μm. (**C**-**D**) NSCLC cells were transfected with shcircPOLK and/or miR-1204 inhibitor for 48 h, and the migratory and invasive abilities of NSCLC cells were determined by Transwell assay. Scale bar: 10 μm. (**E**-**F**) NSCLC cells were transfected with circPOLK and/or miR-1204 mimic for 48 h, and the migratory and invasive abilities of NSCLC cells were determined by Transwell assay. Scale bar: 10 μm

The subsequent investigation focused on elucidating the role of miR-1204 in the progression of NSCLC. As shown in Fig. 5A, miR-1204 inhibitor reinforced the migratory and invasive capabilities of NSCLC cells, and miR-1204 mimic remarkably restrained cell migratory and invasive capabilities (Fig. 5B). These data indicate that miR-1204 is a tumor suppressor in NSCLC progression. Furthermore, miR-1204 inhibitor successfully enhanced the metastatic potential suppressed by shcircPOLK in NSCLC cells (Fig. 5C and D). Meanwhile, miR-1204 mimic reversed the enhancement of metastatic potential induced by circPOLK in NSCLC cells (Fig. 5E and F and Figure S3). Collectively, circPOLK reinforces the metastatic potential of NSCLC cells by miR-1204.

### SOX8 is a downstream target of circPOLK in regulating miR-1204

**Fig. 6:**
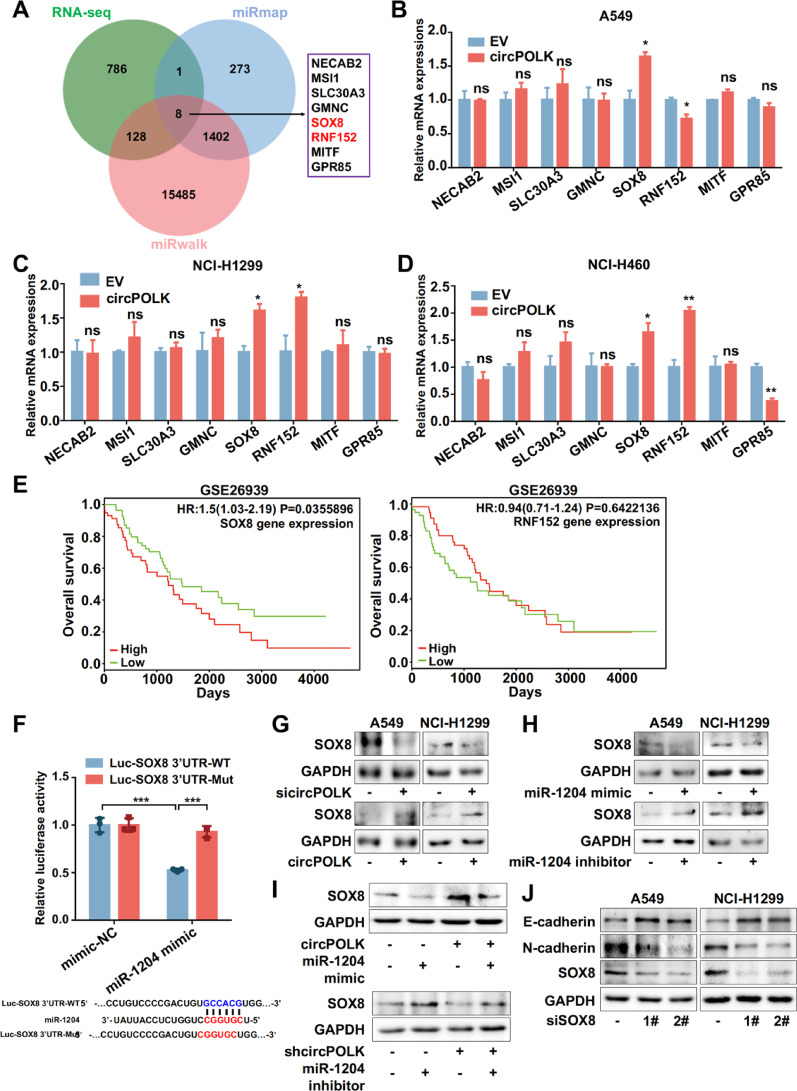
SOX8 is a downstream target of circPOLK in regulating miR-1204. (**A**) A Venn diagram showing the protential target genes of miR-1204 as predicted by miRmap (https://mirmap.ezlab.org/app/) and miRwalk (http://mirwalk.umm.uni-heidelberg.de/) databases and circPOLK regulated DEGs. (**B**-**D**) NSCLC cells were transfected with circPOLK overexpressing plasmid or empty vector for 48 h, and qRT-PCR was used to verify the mRNA level of the predicted targets. (**E**) Survival curves of SOX8 and RNF152 were predicted by PROGgeneV2 database (http://www.progtools.net/gene/). (**F**) The luciferase activities of the SOX8 3’UTR luciferase reporter vector (WT or Mut) were measured after transfection with miR-1204 mimic or mimic NC into NCI-H1299 cells. (**G**) Western blot showing protein levels of SOX8 after overexpressing or silencing of circPOLK in A549 and NCI-H1299 cells. (**H**) Western blot showing protein levels of SOX8 after overexpressing or suppressing of miR-1204 in A549 and NCI-H1299 cells. (**I**) Western blot was performed after transfection with indicated vectors, circPOLK, mimic NC or miR-1204 mimic in NCI-H460 cells. (**J**) Western blot detected the EMT-related protein level of A549 and NCI-H1299 cells after silencing SOX8

We then determined the target proteins of circPOLK/miR-1204, RNA-sequence was used to explore the differentially expressed genes (DEGs) regulated by circPOLK in NSCLC cells (Table S5). Meanwhile, the downstream targets of miR-1204 were predicted by miRmap and miRWalk (Table S6) [41, 42]. A Venn diagram was generated showing that there were eight overlapping genes among circPOLK regulated DEGs, miR-1204 potential downstream targets of miR-1204 predicted by miRmap and miRWalk, including NECAB2, MSI1, SLC30A3, GMNC, SOX8, RNF152, MITF, and GPR85 (Fig. 6A). Then, qRT-PCR was established to further verified the target genes of circPOLK/miR-1204, circPOLK significantly upregulated the expression of SOX8 in three NSCLC cells, while circPOLK downregulated RNF152 in A549 cells and upregulate RNF152 in NCI-H1299 and NCI-H460 cells (Fig. 6B - D). Furthermore, SOX8 overexpression, but not RNF152 overexpression, predicted poor outcomes of lung cancer patients (Fig. 6E) [43]. Thus, we assumed that SOX8 might be a potential target gene of circPOLK/miR-1204. Indeed, miR-1204 mimic could significantly sponge wild type SOX8, but not mutant SOX8 (Fig. 6F). Furthermore, knockdown of circPOLK suppressed the expression of SOX8, and circPOLK promoted SOX8 expression in NSCLC cells (Fig. 6G). Meanwhile, miR-1204 mimic restrained the SOX8 expression, conversely, miR-1204 inhibitor enhanced the level of SOX8 in NSCLC cells (Fig. 6H). More importantly, miR-1204 mimic reversed the expression of SOX8 enhanced by circPOLK, and miR-1204 inhibitor also enhanced SOX8 expression suppressed by shcircPOLK in NSCLC cells (Fig. 6I). In addition, SOX8 silence significantly suppressed the progression of EMT in NSCLC cells (Fig. 6J). Thus, SOX8 might be a downstream target of circPOLK/miR-1204 in regulating NSCLC progression.

### circPOLK promotes angiogenesis via regulating TME

**Fig. 7:**
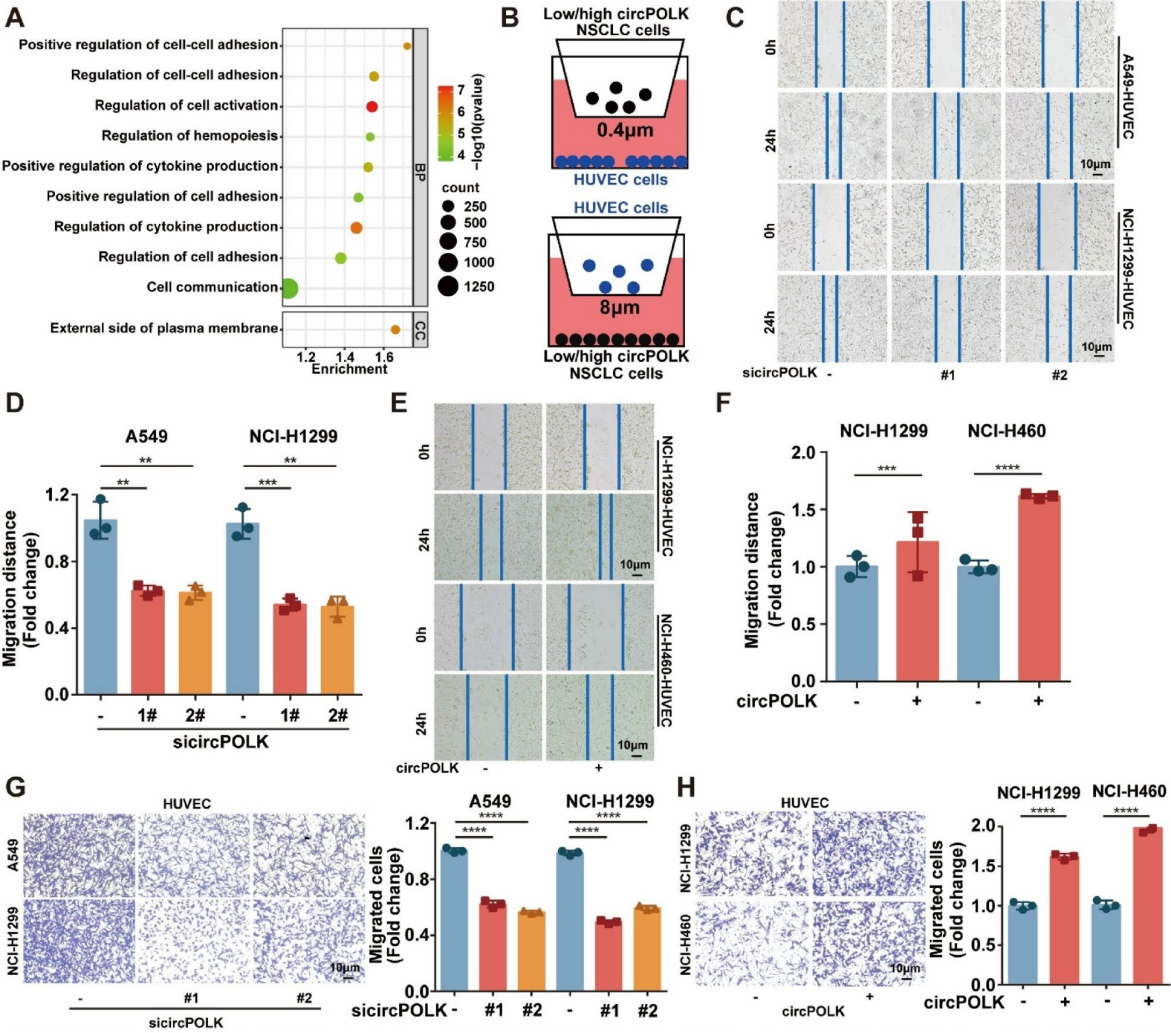
circPOLK promotes angiogenesis via regulating TME. (**A**) GO enrichment of circPOLK regulated DEGs. (**B**, upper panel) The NSCLC cells were cultured on the upper panel of transwell with 0.4 μm pore polyester membrane insert, HUVEC cells were maintained in lower panel of transwell, and wound healing assay was performed to detect migration of HUVEC cells. (**B**, lower panel) The HUVEC cells were cultured on the upper panel of transwell with 8 μm pore polyester membrane insert, NSCLC cells were maintained in lower panel of transwell, and transwell assay was performed to detect migration of HUVEC cells. (**C**-**F**) Wound healing assay detected the migratory abilities of HUVEC cells cocultured with NSCLC cells with circPOLK knockdown or overexpression. (**G**-**H**) Transwell assay verified the migratory abilities of HUVEC cells cocultured with NSCLC cells with circPOLK knockdown or overexpression

GO enrichment of circPOLK regulated DEGs demonstrated that circPOLK might play vital role in cell-cell adhesion and cell adhesion, indicating that circPOLK could participate in the progression of EMT. Furthermore, circPOLK might also be involved in regulation of hemopoiesis and cell activation, cell communication, external side of plasma membrane, and regulation of cytokine production (Fig. 7A and Table S7) [44, 45]. Besides, circPOLK was identified in the exosomes from human blood, we thus hypothesized that circPOLK might be involved in angiogenesis and TME in NSCLC. Then, we constructed coculture systems to mimic angiogenesis in TME of NSCLC (Fig. 7B). NSCLC cells and HUVEC cells were cocultured according to Fig. 7B upper panel, and circPOLK silence in cancer cells could significantly restrain the migratory abilities of HUVEC cells (Fig. 7C and D). In contrast, circPOLK overexpression in NSCLC cells could remarkably enhance the migratory abilities of HUVEC cells (Fig. 7E and F). Meanwhile, we also cocultured NSCLC cells and HUVEC cells as shown in Fig. 7B lower panel, NSCLC cells with circPOLK knockdown restrained the migratory abilities of HUVEC cells in TME, and NSCLC cells with circPOLK overexpression increased the migratory abilities of HUVEC cells (Fig. 7G and H). Thus, the expression of circPOLK in NSCLC cells affected the migratory abilities of vascular endothelial cells in TME, indicating that circPOLK secreted by NSCLC cells might promote angiogenesis of NSCLC.

## Discussion

Approximately 47% of patients diagnosed with NSCLC exhibit metastatic disease upon initial diagnosis, and metastasis is a primary cause of treatment ineffectiveness in NSCLC patients [46, 47]. Nevertheless, the underlying mechanisms of lung cancer metastases are still poorly understood [48]. Tumor-derived angiogenic factors stimulate the migratory and proliferative capabilities of endothelial cells in TME, which leads to angiogenesis supporting NSCLC progression and metastasis [49]. More and more studies have focused on the role of exosomes secreted by lung cancer cells in promoting angiogenesis via cellular communication [50]. In this study, we for the first time verified the expression of circPOLK in human blood exosomes, and circPOLK was a novel circRNA which had never been reported before. Despite the lack of impact on the proliferation of NSCLC cells, circPOLK indeed reinforced metastatic capabilities of NSCLC cells. Furthermore, circPOLK secreted by cancer cells might promote angiogenesis of NSCLC. Thus, this study investigated circPOLK for the first time and its biological function in NSCLC progression and TME, and circPOLK might be a novel therapeutic target for NSCLC patients with metastatic disease. However, the function of exosomal circPOLK in regulating the TME remains poorly understood, and future studies using TME models that mimic angiogenesis will be needed to explore these mechanisms further.

circRNAs frequently serve as competitive endogenous RNAs by sequestering miRNAs, and circRNAs bind to miRNAs and consequently suppress their function [51]. circRNAs function as a miRNA sponge via adsorbing miRNA and combining AGO2 protein, and AGO2 protein acts as a pivotal molecule for the sequestration of miRNA by circRNA [52]. Mechanistically, circPOLK was remarkably enriched by the AGO2 antibody, indicating that circPOLK might promote metastasis of NSCLC cells via sponging miRNAs. We next predicted the potential target miRNAs of circPOLK based on bioinformatics analysis. Combined with RNA pull-down and luciferase reporter assay, we successfully ascertained that miR-1204 was a potential downstream target of circPOLK in NSCLC cells. miR-1204 has been identified as a significant contributor to the development of breast cancer [53]. However, we observed that miR-1204 mimic suppressed the migratory and invasive capabilities of NSCLC cells, and miR-1204 inhibitor enhanced the metastatic potential of NSCLC cells, indicating that miR-1204 has the potential to function as a tumor suppressor in NSCLC. Thus, the biological function of miR-1204 may varied depending on cancer cell type. More importantly, we found that circulating miR-1204 expression was downregulated in the serums of lung cancer patients compared with healthy persons, and low expression of miR-1204 predicted poor outcomes of lung cancer patients, theses data indicated that serumal miR-1204 has the potential to serve as a valuable molecular marker for the diagnosis and prognosis of individuals with lung cancer. Furthermore, miR-1204 mimic reversed the enhancement of migratory and invasive abilities induced by circPOLK, indicating that circPOLK promotes the metastatic potential of NSCLC cells via sponging miR-1204.

Beyond the classical circRNAs framework, recent studies have highlighted the therapeutic relevance of modulating miRNA expression in cancer, including approaches using natural compounds. In addition, RNA modifications-particularly m6A-are increasingly recognized as critical regulators shaping the stability, localization, and functions of non-coding RNAs (including circRNAs and miRNAs) in cancer, and multi-omics strategies are providing new avenues for identifying actionable regulators and facilitating drug discovery [54, 55]. Moreover, emerging evidence suggests that the tumor-microenvironment microbiome may influence cancer progression and therapeutic responses, offering novel perspectives for intervention [56, 57], including potential mechanistic links to immunotherapy-related adverse events [58]. Collectively, these advances broaden the mechanistic and translational context of circRNA-mediated ceRNA regulation and support further exploration of the circPOLK/miR-1204 axis within epitranscriptomic and microenvironmental frameworks.

A circRNA-miRNA-mRNA network was further investigated to verify the target mRNA of circPOLK/miR-1204, and SOX8 was identified as a potential target of circPOLK/miR-1204. SOX8 belongs to SOX transcription factor family members which contain more than 20 members in vertebrates [59]. SOX family member SOX8 has emerged as an important oncogenic regulator in cancer progression. For example, SOX8 was shown to promote chemoresistance in pancreatic cancer by regulating the EZH2/SPARC pathway, and in triple‑negative breast cancer, SOX8 expression is driven by the eEF2K–AURKA axis to enhance tumor growth. These findings support the notion that SOX8 functions as a key driver of malignancy across different tumor types [60, 61]. Furthermore, SOX8 overexpression promotes chemoresistance, cancer stem-like properties and EMT features in tongue squamous cell carcinoma cells [62, 63]. However, the understanding of the involvement of SOX8 in the progression of lung cancer and the direct interaction between miR-1204 and SOX8 remain limited. In this study, we demonstrated that miR-1204 could interact with SOX8 mRNA, and circPOLK could regulate the expression of SOX8 via miR-1204 in NSCLC cells. We also first report that SOX8 regulated the progression of EMT in NSCLC cells, and SOX8 may act as an oncogene in the progression of NSCLC. Previous studies have reported that SOX8 can promote EMT by activating the FZD7‑mediated Wnt/β‑catenin signaling pathway, thereby increasing mesenchymal marker expression (such as N‑cadherin and Vimentin) and suppressing epithelial markers like E‑cadherin, ultimately enhancing metastatic and stem‑like properties of cancer cells [64]. Based on these findings, we speculate that a similar mechanism may also be involved in our model. Although we established the circPOLK/miR‑1204/SOX8 axis, the mechanistic role of SOX8 in driving EMT and metastasis, including its temporal regulation during EMT and potential crosstalk with canonical EMT transcription factors (e.g., SNAIL, SLUG, TWIST, and ZEB1/2), remains to be further investigated and warrants future time‑course studies.

Similar to circRNA-driven ceRNA axes reported in other malignancies (e.g., circRNA_0001573/miR‑382‑5p/FZD3 in colorectal cancer [65] and circRNA–miRNA–mRNA networks in papillary thyroid carcinoma [66], our study identifies an exosome-associated circPOLK/miR‑1204/SOX8 program that promotes EMT, metastasis and angiogenesis in NSCLC. This supports the broader concept that circRNA-mediated miRNA sequestration is a common regulatory mode in cancer progression. Nevertheless, given the pronounced inter- and intra-tumoral heterogeneity of NSCLC and the fact that our conclusions are mainly based on established cell lines and mouse models, further validation in patient-derived specimens/clinical exosomes and more physiologically relevant models will be important to strengthen the clinical relevance of the circPOLK/miR‑1204/SOX8 axis.

Collectively, we first reported the function of circPOLK in cancer progression and angiogenesis. Mechanistically, circPOLK promoted metastasis via miR-1204/SOX8 axis. This work broadens the knowledge of circRNA action in the progression of NSCLC and may have attractive translational potential for NSCLC diagnosis and treatment. Given its pro-metastatic activity, targeting circPOLK may represent a promising adjunct strategy that could be combined with current NSCLC therapies, particularly targeted treatment and immune checkpoint blockade, to potentially enhance therapeutic efficacy and improve clinical outcomes.

## Electronic Supplementary Material

Below is the link to the electronic supplementary material.

Supplementary Material 1

## Data Availability

The data that supports the findings of this study are available in the supplementary material of this article.
