# Supplementary material for: Exosomal circPOLK promotes metastasis of NSCLC cells via regulating mir-1204/SOX8 axis
Source: Cancer Cell Int. 2026 May 8;26:263. doi: 10.1186/s12935-026-04306-2 (PMC13374188; doi:10.1186/s12935-026-04306-2)
Supplement: Supplementary file 1 — Supplementary Material 1 [file 12935_2026_4306_MOESM1_ESM.docx]

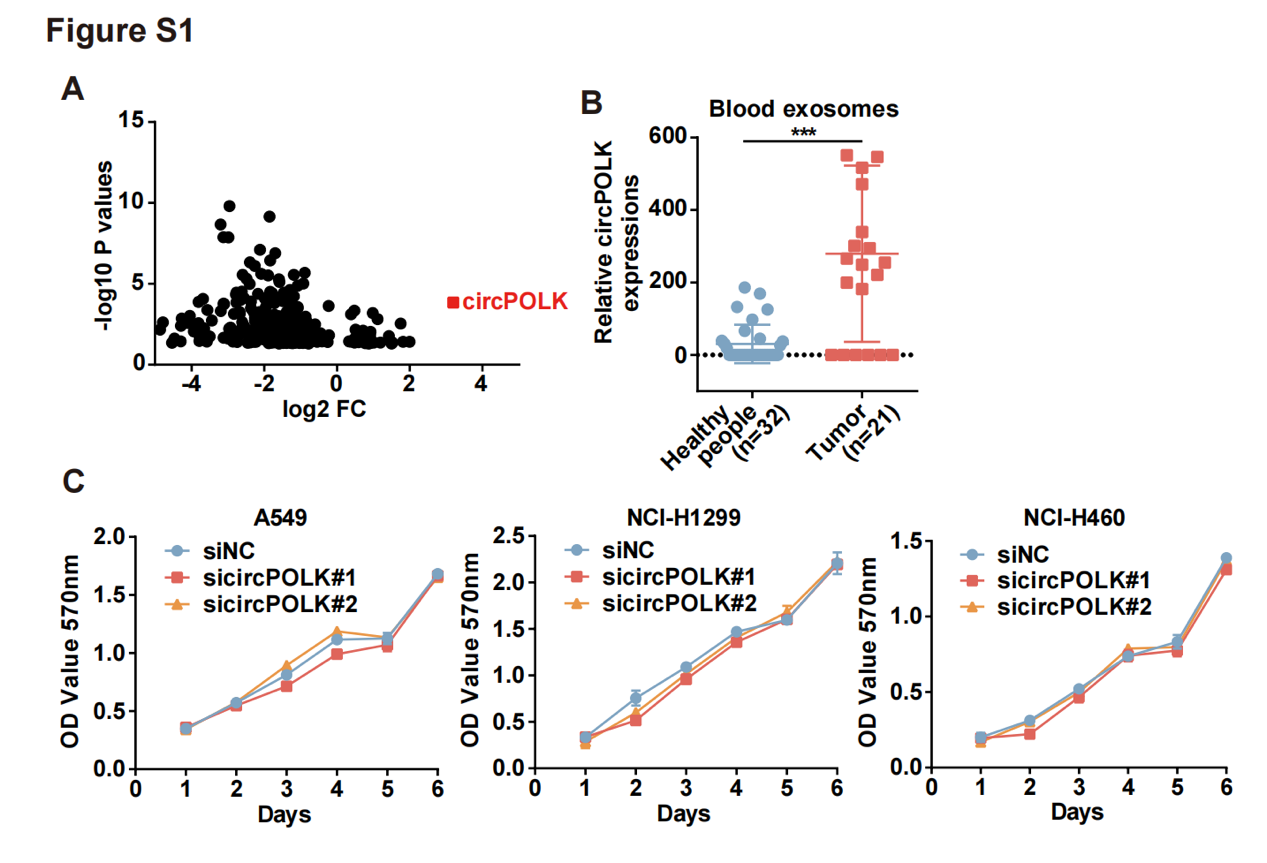


**Figure S1：Analysis of circPOLK expression in blood exosomes and its effect on NSCLC cell proliferation.** (A-B) The data were collected from exoRBase (http://www.exorbase.org/exoRBaseV2/toIndex). Blood exosomes were collected from cancer patients (n=21) and healthy people (n=32). (C) circPOLK could not affect the cell proliferation of NSCLC cells. The effect of circPOLK knockdown on the proliferation of NSCLC cells using SRB assay.


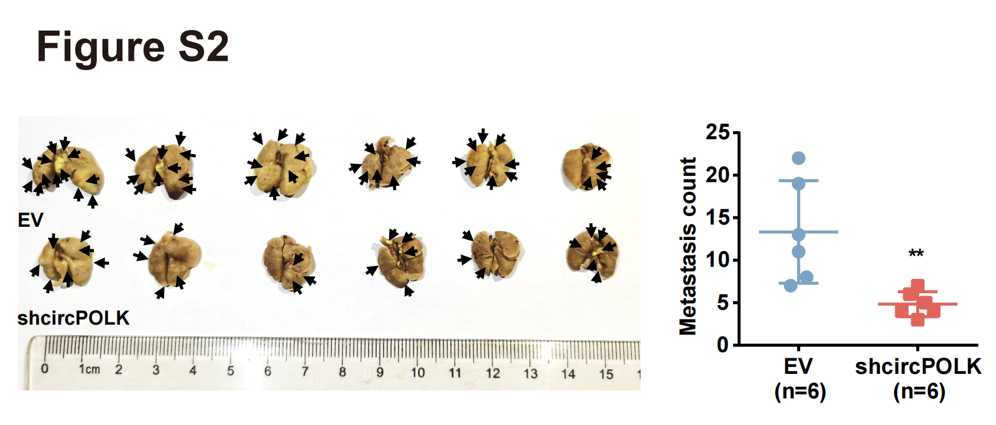


**Figure S2: circPOLK promotes the metastasis of NSCLC cells *in vivo.*** Representative images of lung metastatic tumors (n = 6 / group).


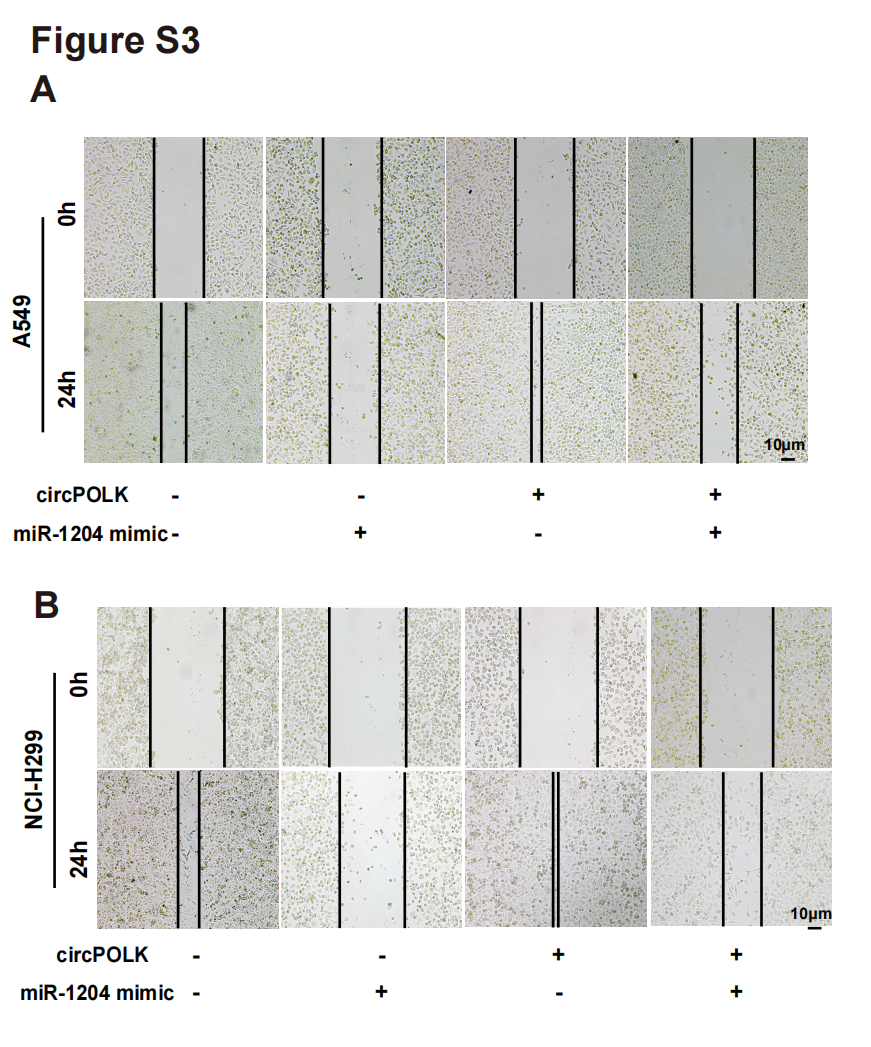


Figure S3. circPOLK enhances the metastatic potential of NSCLC cells through miR‑1204. NSCLC cells were transfected with circPOLK and/or miR‑1204 mimic for 48 h, and their migratory and invasive capacities were assessed using a wound healing assay. Scale bar: 10 μm.
